# Nanoscale Structure Determination of Murray Valley Encephalitis and Powassan Virus Non-Coding RNAs

**DOI:** 10.3390/v12020190

**Published:** 2020-02-08

**Authors:** Tyler Mrozowich, Amy Henrickson, Borries Demeler, Trushar R Patel

**Affiliations:** 1 Department of Chemistry and Biochemistry, Alberta RNA Research and Training Institute, University of Lethbridge, 4401 University Drive, Lethbridge, AB T1K 3M4, Canada; tyler.mrozowich@uleth.ca (T.M.); amy.henrickson@uleth.ca (A.H.); demeler@uleth.ca (B.D.); 2 Department of Chemistry And Biochemistry, University of Montana, Missoula, MT 59812, USA; 3 NorthWest Biophysics Consortium, University of Lethbridge, University of Lethbridge, 4401 University Drive, Lethbridge, AB T1K 3M4, Canada; 4 Department of Microbiology, Immunology and Infectious Disease, Cumming School of Medicine, University of Calgary, Calgary, AB T2N 1N4, Canada; 5 Li Ka Shing Institute of Virology and Discovery Lab, University of Alberta, Edmonton, AB T6G 2E1, Canada

**Keywords:** non-coding RNA, Murray Valley encephalitis virus, Powassan virus, flavivirus, analytical ultracentrifugation, small-angle X-ray scattering, computational RNA structure modeling

## Abstract

Viral infections are responsible for numerous deaths worldwide. Flaviviruses, which contain RNA as their genetic material, are one of the most pathogenic families of viruses. There is an increasing amount of evidence suggesting that their 5’ and 3’ non-coding terminal regions are critical for their survival. Information on their structural features is essential to gain detailed insights into their functions and interactions with host proteins. In this study, the 5’ and 3’ terminal regions of Murray Valley encephalitis virus and Powassan virus were examined using biophysical and computational modeling methods. First, we used size exclusion chromatography and analytical ultracentrifuge methods to investigate the purity of *in-vitro* transcribed RNAs. Next, we employed small-angle X-ray scattering techniques to study solution conformation and low-resolution structures of these RNAs, which suggest that the 3’ terminal regions are highly extended as compared to the 5’ terminal regions for both viruses. Using computational modeling tools, we reconstructed 3-dimensional structures of each RNA fragment and compared them with derived small-angle X-ray scattering low-resolution structures. This approach allowed us to reinforce that the 5’ terminal regions adopt more dynamic structures compared to the mainly double-stranded structures of the 3’ terminal regions.

## 1. Introduction

Family Flaviviridae consists of small, positive-sense single-stranded RNA viruses that replicate within host cells of arthropods and/or vertebrates. Flaviviruses include deadly viruses, such as Murray Valley encephalitis virus (MVEV), Powassan virus (PowV), Japanese encephalitis virus, dengue virus, Zika virus, West Nile virus and yellow fever virus. The World Health Organization and the Centers for Disease Control both cite flaviviruses as a global health threat owing to the ease of transmission by mosquitoes, and the lack of efficient therapeutic or immunoprophylactic strategies [1]. There is a critical need for therapeutics given the magnitude and severity of disease from this class of viruses; however, the limited understanding of the virus’s replication and their complex interactions with the host cellular proteins through terminal region (TR) interactions hinders therapeutic development [2,3,4].

MVEV is a member of the Japanese encephalitis serological complex of flaviviruses and was first isolated in 1951 during the initial outbreak in Australia [5]. It is believed to be contained at the top end of the Northern Territory and the North of Western Australia by a cycle involving mosquitoes (*Culex annulirostris*) and birds [6,7]. The last major outbreak was in 1974 with 58 cases reported, 20% of which resulted in death [8]. There have been increasing instances of MVEV, including nine cases in 2011, three of which resulted in death [9,10]. PowV is the only member of the tick-borne encephalitis serogroup that is currently present in North America [11]. There has been a drastic increase in PowV over the last 18 years as compared to the previous 40 years with an increase of 671% of infections in humans [12]. It was first identified in Powassan, Ontario in 1958 [13] and has become endemic in the upper Midwest and the Northeastern United States, but there have also been cases reported in eastern Russia [14]. The increase in PowV incidences over the last decade highlights the importance of conducting further research to understand the structure-function relationships of the viral genome to ultimately develop therapeutic approaches to combat such viral infections.

Flaviviral genomes consist of a single-stranded RNA molecule approximately 10-11k nucleotides long, depending on species. The RNA genome contains one single open reading frame (ORF), which is flanked by terminal regions (TRs) [1]. The ORF codes for a single polypeptide that is cleaved by a combination of viral and host proteases to produce three structural proteins and seven non-structural proteins [15]. The flanking structural TRs are highly conserved across all flaviviruses and appear as complex folding sequences essential for viral replication [1,16,17,18] (see Supplementary Figure S1). It has been shown that these TRs, which are structurally conserved and intolerable to mutations/deletions, interact with the specific host proteins required for replication [19,20,21]. The important role of these TRs is also highlighted by their ability to cyclize, where the 3’ TR interacts with the 5’ TR [22,23]. This cyclization of the viral genome is essential for the correct positioning of the NS5 RNA dependent RNA polymerase produced by the viral genome [24]. NS5 is required in order to produce the (-) sense RNA that is used as the replication template for further production of the viral (+) sense RNA genome [25]. Flaviviral TRs also interact and are recognized by a variety of host proteins [22,26,27], including the host innate immune sensor; 2’ 5’ oligoadenylate synthetase (OAS)-family proteins and RNA helicases (DDX3X, DDX5, and DDX6) [4,20,26,27,28,29]. To further understand viral replication, it is essential to study not only the complex secondary and tertiary structures of viral TRs, but also how their structure influences their interactions with host proteins and cyclization events. For example, previous studies on the interactions of West Nile virus and adenoviral RNA with host innate immune system proteins provided insights into the RNA-protein interaction, as well as mutation dependent structural changes on adenoviral RNA [22,26,30,31,32]. 

Although several attempts have been made to study RNA structures in general [33,34], detailed insight into the structural features of multiple flaviviral strains is lacking. Such insights could provide a platform to develop novel antiviral therapies as well as further our knowledge about viral replication events. Using a combination of biophysical techniques and computational calculations, we constructed 3-dimensional structures for the 5’ and 3’ TRs of both MVEV and PowV. This study provides insight into the structural organization of flaviviral TRs and provides a framework for the characterization of other flaviviral TRs, or TR segments or other non-coding RNAs.

## 2. Materials and Methods 

### 2.1. RNA Preparation and Purification

We prepared cDNA sequences under the control of a T7 RNA polymerase promoter with two additional G nucleotides on the 5’ end followed by an XbaI restriction enzyme cut site (T^CTAGA). We designed our constructs for MVEV and PowV based on the Genebank sequences of KX229766.1and EU670438.1, respectively. All RNA constructs used in the experiments are listed as follows:

1. MVEV 5TR 1-96nt
  5’GGAGACGUUCAUCUGCGUGAGCUUCCGAUCUCAGUAUUGUUUGGAAGGAUCAUUGAUUAACGCGGUUUGAACAGUUUUUUGGAGCUUUUGAUUUCAAU3’
2. MVEV 3TR 10914-11014
  5’GGCCUGGGAAAAGACUAGGAGAUCUUCUGCUCUAUUCCAACAUCAGUCACAAGGCACCGAGCGCCGAACACUGUGACUGAUGGGGGAGAAGACCACAGGAUCUU3’
3. POWV 5TR 1-111
  5’GGAGAUUUUCUUGCACGUGUGUGCGGGUGCUUUAGUCAGUGUCCGCAGCGUUCUGUUGAACGUGAGUGUGUUGAGAAAAAGACAGCUUAGGAGAACAAGAGCUGGGAGUGGUUU3’
4. POWV 3TR 10735-10839
  5’GGCCCCCAGGAAACUGGGGGGGCGGUUCUUGUUCUCCCUGAGCCACCACCAUCCAGGCACAGAUAGCCUGACAAGGAGAUGGUGUGUGACUCGGAAAAACACCCGCUU3’

We performed in vitro transcription reactions using T7 RNA polymerase to prepare each RNA, followed by their purification using a Superdex 200 increase (GE Healthcare Canada inc, Mississauga, ON, Canada) on an ÄKTA pure FPLC (fast protein liquid chromatography) system (GE Healthcare) at 0.5mL/min. We collected and pooled fractions containing purified RNAs from the size exclusion chromatography (SEC) fraction collector. Pooled fractions were ethanol precipitated and resuspended in RNA buffer (50 mM Tris pH 7.5, 100 mM NaCl and 5 mM MgCl_2_). Next, we analyzed the pooled fractions using Urea-Polyacrylamide Gel Electrophoresis (Urea-PAGE). We mixed 10 µL of ~500 nM RNA with 2 µL of RNA-loading dye and placed them into a 1.0 cm well PAGE casting plate (Bio-Rad Laboratories (Canada), Mississauga, ON). Finally, we ran the Urea-PAGE (7.5%) at 300V at room temperature for 25 minutes in 0.5 X TBE, followed by staining with Sybr Safe (Thermofisher Scientific, Saint-Laurant, QC) and UV visualization. Prior to performing analytical ultracentrifugation (AUC) and small-angle X-ray scattering (SAXS) experiments, we heated RNA to 95 °C for 5 min and cooled passively to room temperature to facilitate refolding.

### 2.2. Analytical Ultracentrifugation (AUC)

The sedimentation velocity (SV) - AUC data for FPLC-purified RNA samples were collected using a Beckman Optima AUC centrifuge and an AN50-Ti rotor at 20 °C. We loaded MVEV 5’ (0.6 uM), MVEV 3’ (0.5 uM), PowV 5’ (0.6 uM) and PowV 3’ (0.58 uM) samples into Epon-2 channel centerpieces in 50 mM Tris pH 7.5, 100 mM NaCl, and 5 mM MgCl_2_ buffer. As a first step, we centrifuged samples at 35,000 revolutions per minute and collected scans at 20-second intervals. We used the UltraScan-III package [35] to analyze all data using Comet (San Diego Supercomputing Center, San Diego, CA) and Lonestar5 (Texas Advanced Computing Center, Austin, TX). We initially analyzed the SV-AUC data using two-dimensional spectrum analysis (2DSA) with simultaneous removal of time-invariant noise, meniscus and bottom positions fitted [36], followed by enhanced van Holde - Weischet analysis [37] and genetic algorithm refinement [38]. We derived fitting statistics from the Monte Carlo analysis [39] and estimated the buffer density and viscosity corrections with UltraScan (1.0030g/cm3 and 1.0100 cP, respectively). All hydrodynamic parameters were corrected to standard conditions at 20 °C and water.

### 2.3. Small-Angle X-ray Scattering (SAXS)

We utilized the B21 beamline at Diamond Light Source (Didcot, Oxfordshire, UK) to collect small-angle X-ray scattering (HPLC-SAXS) data as previously described [40]. Making use of an in-line Agilent 1200 (Agilent Technologies, Stockport, UK) HPLC connected to a flow cell, each purified RNA, 50 µL of ~2.0 mg mL^−1^ was injected into a buffer equilibrated Shodex KW403-4F (Showa Denko America Inc., New York, NY, USA) size exclusion column at a flow rate of 0.160 mL per minute. Each frame was exposed to the X-rays for 3 seconds. Each sample peak region was buffer subtracted and merged using Primus [41] or ScÅtter [42] as described previously [43]. We analyzed the merged data using Guinier approximation to obtain the radius of gyration (*R_g_*) and study homogeneity of samples [44]. We also performed dimensionless Kratky analysis [45] to investigate if the RNA molecules of interest are folded, as reviewed earlier [46]. Next, we performed the pair-distance distribution (*P*(*r*)) analysis using the program GNOM [47], which provided the *R_g_* and the maximum particle dimension (*D_max_*, the radius at which the *P*(*r*) dependence approaches zero). We used information from the *P*(*r*) plot to generate the models using DAMMIN [48], with no enforced symmetry, as described previously [49]. Lastly, we averaged and filtered the resulting models to obtain a singular representative model using the DAMAVER package [50], as described previously [31,51].

### 2.4. Atomic Structures Calculations

**Figure 1:**
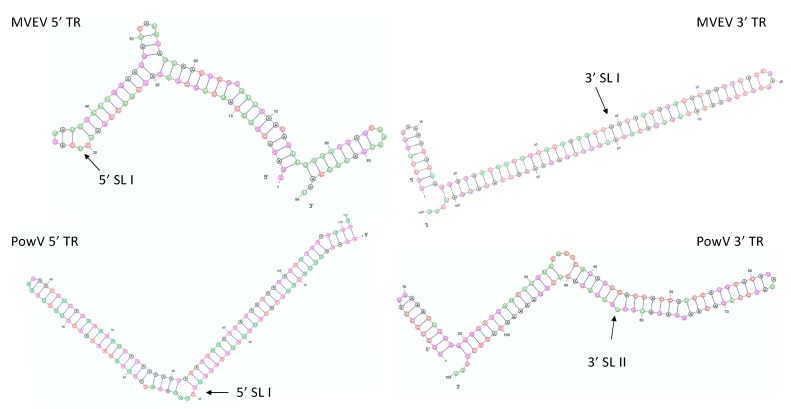
Lowest energy predicted secondary structures of Murray Valley encephalitis virus (MVEV) and Powassan virus (PowV) non-coding RNA regions visualized using VARNA [55]. The next three lowest energy structures are presented in Supplementary Figure S2. The arrows represent stem-loop I (SLI) location in 5’ and 3’ TRs.

To generate secondary structure predictions used downstream, we used MC-Fold [52] to predict numerous low energy secondary structures for 5’ and 3’ TRs of MVEV and PowV (Figure 1). We selected the lowest energy structures in each case as input files for MC-Sym that enables the fragment-based reconstruction of 3-dimensional structures using known structures, as described earlier [52]. Through MC-Sym, we calculated 100 all-atom structures for each RNA and minimized using protocols implemented in MC-Sym. Next, we simulated scattering profiles for each MC-Sym calculated structure with CRYSOL based on minimized structures and compared against experimental SAXS data. We subjected the minimized structures to CRYSOL to determine *R_g_* and the goodness-of-fit parameter (*χ^2^*) [53]. Finally, we ranked the MC-Sym derived structures based on their *χ^2^* values and aligned them with low-resolution structures using the program SUPCOMB [54]. 

## 3. Results

### 3.1. Purification of In-Vitro Transcribed RNA

**Figure 2:**
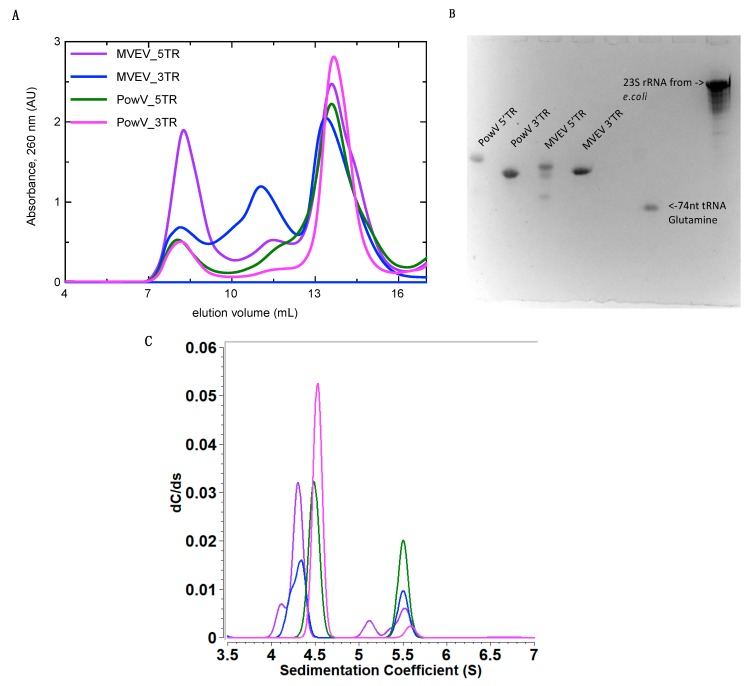
Purification and characterization of in-vitro transcribed 5’ and 3’ terminal regions (TRs) of MVEV and PowV RNA. (**A**) Size exclusion chromatography (SEC) elution profile of purified RNAs where the x-axis represents elution volume and the y-axis represents absorbance at 260 nm; (**B**) Urea-PAGE demonstrating the purity of the individual pooled RNA fractions from SEC peaks (~13 to 14.5 mL). For comparison purposes, we have included 74 nts tRNA and 23S rRNA from *Escherichia coli* (**C**) Sedimentation coefficient distribution profiles of MVEV and PowV non-coding TRs obtained from sedimentation velocity-analytical ultracentrifugation (SV-AUC). The SV peaks at ~4.5 S for each RNA represent monomeric fractions. The sedimentation coefficient values are corrected to standard solvent conditions (20 °C in water).

We purified the in-vitro transcribed RNAs using a Superdex 200 increase column connected to the ÄKTA FPLC unit. Figure 2A presents an elution profile with all four RNA constructs overlayed, where peaks at ~8 mL represent elution of template plasmids used to in-vitro transcribe these RNAs. The RNAs of interest eluted at ~13 to 14.5 mL and the peaks at ~11 mL suggest the presence of an oligomeric assembly of RNAs. After SEC, we pooled the monodispersed fractions (between ~13 to 14.5 mL) for each RNA and analyzed them using Urea-PAGE (Figure 2B). As demonstrated in Figure 2B, RNAs migrate similarly and are highly pure, devoid of any aggregation or degradation, except minor degradation of MVEV 5’ TR. These fractions were stored at 4 °C until further experiments were carried out.

### 3.2. Homogeneity Studies of RNA 

Analytical ultracentrifugation is a versatile technique to study the purity of biomolecules in solution [56]. In an AUC experiment, biomolecules are subjected to a high centrifugal force (up to 250,000 × g) to separate them on the basis of their size, anisotropy and density. The separation of biomolecules is monitored by means of an optical system. To investigate the purity of all four RNAs, we performed SV experiments at concentrations ranging between 0.5 and 0.7 µM and processed the data using UltraScan [35] as described in the Materials and Methods section. Figure 2 (C) presents the sedimentation coefficient distribution for MVEV 5’ and 3’ as well as PowV 5’ and 3’ RNA fragments. The SV analysis suggests that all four RNAs are mainly monodisperse with sedimentation coefficient values of 4.27 S for MVEV 5’ TR, 4.30 S for MVEV 3’TR, 4.49 S for PowV 5’ TR and 4.53 S for PowV 3’ TR (S = 10^−13^ seconds, s values corrected to 20 °C for water), as summarized in Table 1. Note that the peaks at ~ 5.5 S suggest that all four RNAs form dimeric or higher-order conformations in solution. The AUC data also suggest that PowV TRs with a M_w_ of ~38 kDa have a slightly higher sedimentation coefficient compared to the MVEV TRs with M_w_ of ~32 kDa (Table 1). Overall, these experiments indicate that all four RNAs are of suitable purity to perform HPLC-SAXS to determine the solution structure. 

### 3.3. Low-Resolution Structural Studies of RNAs

**Figure 3:**
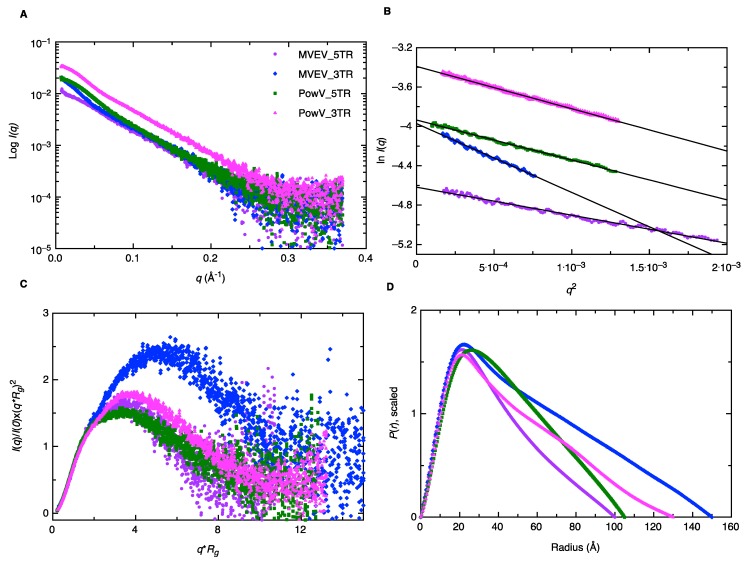
Characterization of MVEV and PowV terminal regions using small-angle X-ray scattering (SAXS). (**A**) A plot of scattering intensity (Log *I*(*q*)) versus scattering angle (*q* = 4πsinθ/λ) representing merged SAXS data for MVEV and PowV. (**B**) Guinier plots (plot of *ln*(*I*(*q*)) versus *q^2^*) representing the homogeneity of samples and allowing determination of *R_g_* based on the low-angle region data. (**C**) Dimensionless Kratky plots (*I*(*q*)/*I*(*0*)*(q*R_g_)^2^ vs q*R_g_) for all four RNA samples demonstrating their extended structures. (**D**) Pair-distance distribution (*P*(*r*)) plots for all four RNA samples representing their maximal particle dimensions and allowing the determination of *R_g_* from the entire SAXS dataset.

Small-angle X-ray scattering allows for structural determination of biomolecules and their complexes under physiological conditions, albeit at low-resolution. Recent improvements in instrumentation employ an HPLC unit connected in line with SAXS detection to improve the monodispersity of samples and to resolve species of interest from aggregated and degraded products [57,58]. This advancement allows us to be confident in the monodispersity of our samples, even when minor heterogeneity is present. As outlined in the Materials and Methods section, we collected HPLC-SAXS data for all four RNAs, followed by the selection of data from a monodisperse peak, buffer-subtraction, and merging of selected datasets. The merged SEC-SAXS data are presented in Figure 3A. Subsequently, we processed the merged data using the Guinier method (plot of (*I*(*q*)) vs. (*q^2^*)), which aids detection of purity and allows determination of the *R_g_* (average root mean squared radius from the center of the mass for a biomolecule) from the data belonging to the low-*q* region [44]. Figure 3B presents the Guinier plots for 5’ and 3’ TRs of MVEV and PowV, where the linearity for low-*q* data demonstrates that all four RNAs are monodispersed and devoid of any aggregation. Based on the Guinier analysis, we obtained *R_g_* values of 29.01 ± 0.22 Å, 45.72 ± 0.48 Å, 34.65 ± 0.19 Å and 35.84 ± 0.13 Å for MVEV 5’ TR, MVEV 3’ TR, PowV 5’ TR and PowV 3’ TR respectively (see Table 1 for more details). Once we confirmed the monodispersity, we processed the SAXS scattering data from Figure 3A to obtain dimensionless Kratky plots (*I*(*q*)/*I*(*0*)*(q*R_g_)^2^ vs q*R_g_) that allow detection of the folding state of biomolecules [46,58]. For example, globular-shaped biomolecules in solution are observed with a well-defined maximum value of 1.1 at q*R_g_ = 1.73 [45]. The dimensionless Kratky plots for 5’ and 3’ TR of MVEV and PowV under investigation demonstrate that all the samples are well folded, and extended in solution (Figure 3C).

Next, using program GNOM [47], we performed an indirect Fourier transformation to convert the reciprocal-space information (*ln*(*I*(*q*)) vs. (*q*), Figure 3A) into the real space electron pair-distance distribution function (*P*(*r*), Figure 3D) to obtain *R_g_* and *D_max_* (maximum particle dimension) for all four RNAs. It is important to note that unlike Guinier analysis, which is restricted to the data in the low-*q* region, the *P*(*r*) analysis utilizes a wider-range of the dataset and aids in a reliable determination of *R_g_* and *D_max_*. As outlined in Table 1, based on *P*(*r*) analysis, we obtained the *D_max_* of 100 Å, 150 Å, 105 Å, and 130 Å for MVEV 5’ TR, MVEV 3’ TR, PowV 5’ TR, and PowV 3’ TR respectively. Furthermore, we obtained the *R_g_* values of 30.16 ± 0.11 Å, 46.16 ± 0.18 Å, 34.48 ± 0.06 Å and 38.39 ± 0.08 Å for MVEV 5’ TR, MVEV 3’ TR, PowV 5’ TR and PowV 3’ TR respectively. These values are very similar to those obtained from Guinier analysis, which indicates that the data are suitable for low-resolution shape reconstruction. The shape of the *P*(*r*) plot is indicative of the solution conformation of biomolecules. For example, we would have expected a bell-shaped *P*(*r*) distribution curve with a maximum at ~*D_max_*/2 [59] for a globular-shaped protein; however, all four RNAs display skewed bell-shaped curves with extended tails that suggest their extended structures in solution as is shown in Figure 3D.

**Figure 4:**
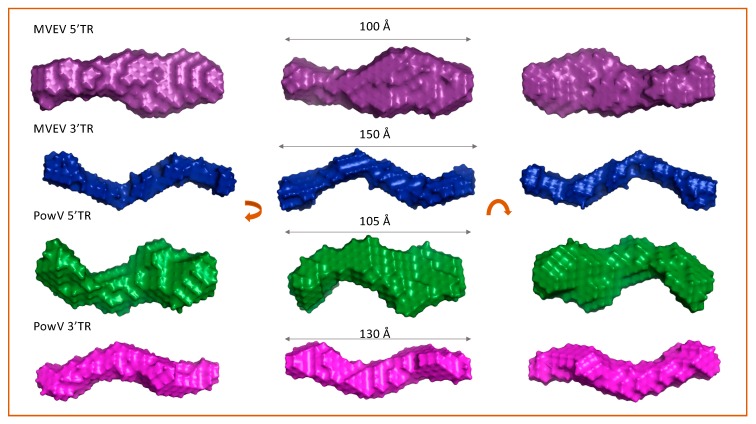
Determination of low-resolution structures using SAXS, which indicates that all RNA molecules adopt an extended structure in solution. For each terminal region RNA, the left and right panels represent 180° rotated view on the x-axis and y-axis, respective of the view presented in the middle panel. Dimensions represent the *D_max_*.

To obtain low-resolution structures for each RNA, we employed DAMMIN [48] that utilizes a simulated-annealing protocol and allows the incorporation of *P*(*r*) data (i.e., *D_max_* and *R_g_* as constraints). We calculated a total of 12 models for 5’ and 3’ TRs of MVEV and PowV and noted that individual models had an excellent agreement between the experimentally obtained scattering data and the calculated scattering data. The χ^2^ values in each case are ~0.8, which represents an agreement between the experimentally collected scattering data and the low-resolution model derived scattering data (see Table 1). Next, we employed program DAMAVER [50] to rotate and align all 12 models and to obtain an averaged filtered structure for each RNA, that represents structural features from individual models (Figure 4) [50]. In each case, the goodness of the superimposition of individual models was estimated by the overlap function—the normalized spatial discrepancy (NSD). As presented in Table 1, the low NSD values suggest that 12 models in each case are highly similar to each other. Figure 4 presents the averaged filtered structures for the 5’ and 3’ TRs of MVEV and PowV, which indicates that, overall, these RNAs have extended structures in solution. 

### 3.4. Computational Modeling of RNA Structures 

**Figure 5:**
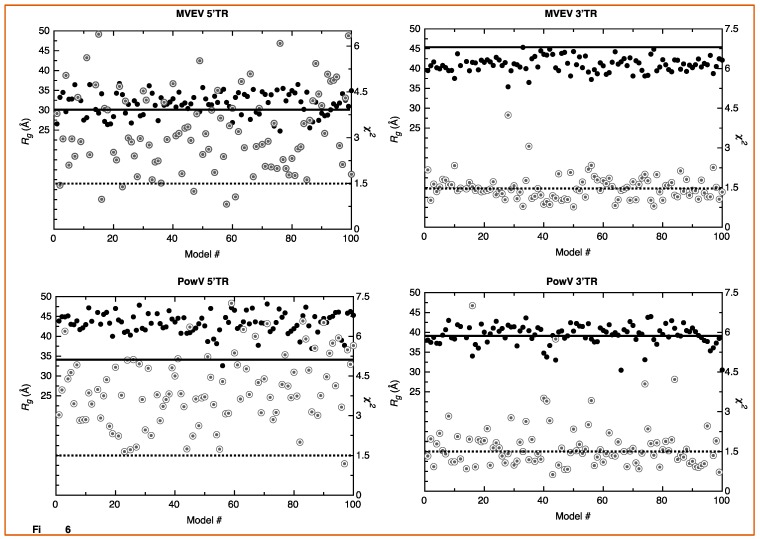
Screening of high-resolution structures calculated using MC-Sym [52] for MVEV 5’ TR, MVEV 3’TR, PowV 5’TR and PowV 3’TR. For each RNA, the x-axis represents model numbers (total 100 models), whereas the y1-axis (solid black circles) and y2-axis (grey double circles) represent *R_g_* (Å) and χ^2^ values, respectively, calculated using CRYSOL package [53]. For each plot, the solid dark line corresponds to y1-axis and represents the value for experimentally determined *R_g_* (Å) as presented in Table 1. The dotted line corresponds to y2-axis and represents a χ^2^ value of 1.5. These plots indicate that MC-Sym derived structures represent a wide-range of conformations these RNAs can theoretically adapt.

SAXS is an outstanding method that allows the determination of solution structures of biomolecules, albeit at low-resolution. Techniques such as X-ray crystallography and nuclear magnetic resonance spectroscopy provide high-resolution structural information but have limited applications for RNA structural studies due to challenges obtaining high-quality crystals and RNA-labeling, respectively. An alternative to this approach is to employ computational modeling of RNA structures and screen those structures using experimental data. We employed the MC-Fold/MC-Sym pipeline developed by Parisien and Major [52]. For each RNA, we calculated 100 structures using the MC-Sym pipeline based on the secondary structure predicted using MC-Fold (Figure 1). The ΔG values for the lowest energy structures for MVEV 5’TR, MVEV 3’TR, PowV 5’TR, and PowV 3’TR are −86.41, −104.44, −103.07, and −103.07 kcal mol−1, respectively. Next, we used program CRYSOL [53] to calculate X-ray scattering profiles of 100 structures of each RNA (5’ and 3’ TRs of MVEV and PowV) and compared these data with experimentally collected X-ray scattering data to calculate χ^2^ value. We also determined *R_g_* from MC-Sym derived structures. Figure 5 presents the *R_g_* (y-axis left-hand side, solid black circles) and χ^2^ values (y-axis right-hand side, grey double circles) for each RNA system, which suggests that the RNA molecules under investigation can adopt a variety of conformations. For example, in the case of 5’ TR of MVEV, the distribution of *R_g_* values range from 30 ± 5 Å, whereas the experimentally determined *R_g_* for this construct is 30.16 Å. Similarly, for PowV 3’ TR, experimentally determined *R_g_* is 38.39 Å, which largely aligns with the distribution of *R_g_* values of MC-Sym derived structures. On the other hand, the MC-Sym derived models for MVEV 3’ TR mainly under-represent the *R_g_* values when compared to experimental *R_g_* of 46.16 Å. In contrast, for PowV 5’TR, Mc-Sym derived structures have a higher *R_g_* distribution (~45Å) compared to the experimentally determined *R_g_* (34.48 Å). Moreover, as presented in Figure 5, the χ^2^ values for MC-Sym derived 3’ TR of MVEV and PowV structures have better distribution around 1.5 χ^2^, compared to MVEV and PowV 5’ TRs. 

### 3.5. Combination of Computational Modeling and Experimental Low-Resolution SAXS Structures

**Figure 6:**
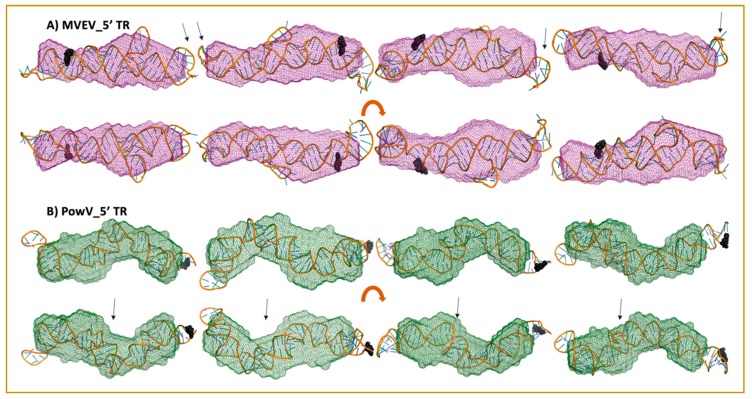
High-resolution structures calculated using MC-SYM overlaid with low-resolution SAXS models. A) MVEV 5TR and B) PowV 5TR. Bottom panels in both cases represent a 180^o^ rotation along the x-axis represented in the top panels. Black spheres represent a 5’ terminal region on each construct. The arrows represent stem-loop I (SLI) location in 5’ TRs.

**Figure 7:**
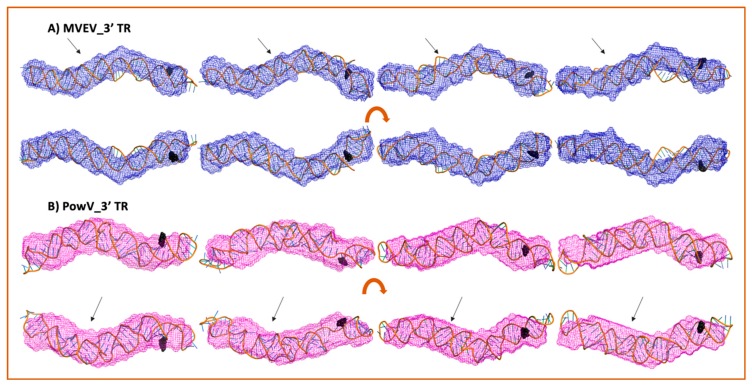
High-resolution structures calculated using MC-SYM overlaid with low-resolution SAXS models. A) MVEV 3TR and B) PowV 3TR. Bottom panels in both cases represent an 180^o^ rotation along the x-axis represented in the top panels. Black spheres represent 5’ terminal region on each construct. The arrows represent stem-loop I (SLI) location in 3’ TRs.

We selected ~10 MC-Sym derived structures with the lowest χ^2^ values to further investigate if they align with the low-resolution averaged filtered models for each RNA system. Figure 6 and Figure 7 present four of the best χ^2^ fit MC-Sym derived structures for MVEV and PowV, indicating that these structures agree well with the low-resolution models based on SAXS data. Overall, MC-Sym derived structures for the 3’ TRs of MVEV and PowV (Figure 7) indicate that they adopt highly extended structures mainly in the double-stranded regions. In contrast, the 5’ TRs of MVEV and PowV are more dynamic with less double-stranded regions (Figure 6). Furthermore, these results indicate that the 5’ TR structures have a higher content of un-paired/loop regions compared to the 3’ TRs which contains mainly double-stranded regions. 

## 4. Discussion

MVEV is endemic to northern Australia and has seen an increase in prevalence during the last decade. The likelihood of an outbreak is increasing given the extensive human development in affected areas. PowV is an under-studied flavivirus despite its increasing incidence and severe neurological effects. Patients infected with almost any flavivirus, including PowV and MVEV, are treated only with symptomatic support. Treatment of the viral infection is nonexistent. Further investigation into these potentially deadly viruses is necessary to gain detailed insights into the viral life cycle in order to prepare ourselves better for potential future outbreaks.

The lowest energy secondary structure predictions (Figure 1) reveal that each RNA molecule varies slightly due to increasing energy, except for PowV 5’ TR and to a lesser degree MVEV 3’ TR. The structure with the lowest predicted energy of PowV 5’ TR displays a large change in secondary structure with only a small change in energy (−103.07 vs. −102.43). The secondary structure information for MVEV 3’ TR indicates that a small increase in energy could result in the formation of a second stem-loop with a small energy change (−104.44 to −103.29, Supplementary Figure S2). This suggests that the RNA molecules can adopt different conformations in solution.

We prepared all four RNAs using in-vitro transcription with T7 polymerase and purified using SEC (Figure 2B), which indicates that these RNAs can form dimer/oligomers in solution. The fractions indicating a homogenous preparation were pooled together and analyzed using Urea-PAGE (Figure 2B), where the three RNAs displayed a single band, and MVEV 5’TR showed a slight degradation. We used an RNA that was smaller than our constructs (74nt tRNA), and another, which was larger, (23S rRNA) to represent a relative difference in RNA size (Figure 2B), (Table 1). We further employed SV experiments using analytical ultracentrifugation to determine the composition and anisotropy of biomolecules in solution [56,61,62]. Figure 2 presents the sedimentation coefficient distribution of SEC-purified RNA preparation. As evident by the peaks between 5S and 6S, the RNAs can form higher-order oligomers to a small degree despite removing many of them by means of SEC purification. This is consistent with the HPLC data from SAXS, which also shows a minor amount of oligomerization for each RNA. An explanation for these minor oligomerization peaks could be the RNA adopting different conformations, as seen in the predicted secondary structure(s) of PowV 5’ TR (Supplementary Figure S2) where a small energy change caused the RNA to adopt a different conformation. This prediction could explain the increased peak that is evident at 5.5 S (Figure 2C) for both PowV 5’ TR and MVEV 3’ TR. Additionally, the peak at ~ 4 S for MVEV 5’ TR indicates minor degradation, which was also seen in the Urea-PAGE (Figure 2B,C). More importantly, the differences in the RNA size and shape are indicated by their sedimentation coefficient values (Table 1). Both the TRs of MVEV have a *M_w_* of ~ 32 kDa and produce very similar S values (4.27 S and 4.3 S, for 5’ and 3’ TR, respectively). Furthermore, the S values for 5’ and 3’ TRs of PowV are 4.50 S and 4.53 S. This similarity correlates with the similarities of their *M_w_* of ~ 37 kDa (Table 1).

SAXS is an excellent complementary structural-biophysical method, which enables solution structural studies of RNA, proteins, and their complexes, albeit at low-resolution [34,46,49,57,63,64,65]. Solution X-ray scattering is employed for biological systems where obtaining high-quality crystals or labeling of biomolecules presents challenges [22,30,31,49,63,64]. In this study, we employed an HPLC-SAXS set-up to collect scattering data from a preparation free of aggregation or degradation. The monodispersed preparation was confirmed by Guinier analysis and shows excellent linearity of fit in the low-*q* region (Figure 3B). Furthermore, Guinier analysis also provided *R_g_* of all four RNAs (based on the low-*q* region), which are highly similar to those calculated by means of *P*(*r*) analysis (Figure 3D, Table 1). We also performed dimensionless Kratky analysis (Figure 3C) that demonstrates each RNA adopts an elongated structure, with a low amount of flexibility. The *P*(*r*) distribution reveals a quick increase to the highest point and then a gradual decrease where the electron-pair-distance approaches zero. A globular molecule would result in a Gaussian-like distribution, which is not evident in any of the RNA, and suggests their elongated nature. The most extended molecule is MVEV 3’ TR resulting in a *D_max_* of 150 Å, even though its molecular weight is 31.57kDa (Table 1), which is lower than both 5’ and 3’ TR of PowV (Table 1). Low-resolution structure modeling (Figure 4) confirms that MVEV 3’ TR adopts a much thinner and elongated structure, which suggests that this RNA is almost entirely helical. This is also evident for PowV 3’ TR, which also displays an extended conformation. This elongation is consistent with the predicted secondary structure of flaviviral 3’ terminal regions where it is suggested the last ~100 nucleotides almost entirely base pair into a large system [22,66,67,68]. Low-resolution structures of 5’ TRs of MVEV and PowV are less elongated when compared to the 3’ TRs; however, their low-resolution structures appear to be consistent with the predicted secondary structures of flaviviral 5’ TRs. This indicates that the 5’ TRs adopt more dynamic structures [26,66,67,68]. Overall, MVEV and PowV TR structures are consistent with previously published low-resolution structures of flaviviral RNA such as the Zika and West Nile viruses [22,26,69]. Although the flaviviruses possess a methylated type 1 cap (me7-GpppA-me2) on the 5’ terminus, our in vitro transcribed RNAs are not methylated, and we anticipate that an addition of an extra guanine residue through a non-standard 5’ to 5’ triphosphate linkage would not affect the low-resolution structure determination. Furthermore, due to T7 requiring a guanine residue to start transcription, our constructs actually contain a G nucleotide on the 5’ end similar to a type 1 cap.

A strength of SAXS is its ability to be combined with high-resolution structures/homology models of individual domains, as well as with computational studies to complement predicted structures [22,30,46,49,63]. RNA structures of high resolution are hard to determine through crystallization and labeling studies mentioned above; therefore, an attractive alternative to calculating high-resolution structures is computational modeling. To this end, we employed the MC-Fold/MC-SYM pipeline to reconstruct all-atom structures and screen those using experimentally collected X-ray scattering data to identify structures that are likely adopted by these RNAs. For each TR structure, we used CRYSOL [53] to calculate *R_g_* as well as back-calculated X-ray scattering data. We aligned this data with experimentally collected scattering data (Figure 5). This analysis demonstrates that MVEV 3’ TR and PowV 3’ TR exhibit a strong correlation between the experimentally determined *R_g_* and the calculated *R_g_* from MC-Sym-derived structures. For both of these RNAs the *χ^2^* values indicate agreement between scattering data that were experimentally collected and calculated from MC-Sym-derived structures, where the *χ^2^* values were close to ~1.5 for most structures (Figure 5). On the other hand, the *χ^2^* values for MC-Sym-derived structures for 5’ TRs of MVEV and PowV have a much larger distribution (from ~1.5 to ~6). A similar trend was also observed for *R_g_* for both 5’ TRs where the MC-Sym derived structures displayed a wider distribution of *Rg*, suggesting that these RNAs could adopt multiple conformations. The correlation between *R_g_* and *χ^2^* was far more pronounced in the more elongated TR RNAs - PowV 3’ TR and MVEV 3’ TR (Figure 4 and Figure 5). Finally, we selected the ~10 MC-Sym-derived structures, which had the lowest *χ^2^* values when compared to experimental data and aligned them into the low-resolution SAXS envelopes. Selected groups of structures are presented in Figure 6 and Figure 7, which indicate that the computationally calculated structures agree well with the experimental data, especially for the 3’ TRs. For the 5’ TRs, we observed dynamic structures; however, they maintain a similar conformation in solution overall. Furthermore, the bulge regions in both the 5’ TRs also agree well with their secondary structures, which suggests that these secondary structures could be conserved and do not undergo conformational changes. An added benefit of these computationally derived structures is the addition of directionality of the RNAs (the black spheres represent the 5’ end of each molecule in Figure 6 and Figure 7), which is otherwise very challenging to decipher unless the molecule of interest is altered [70].

## 5. Conclusions

In this study, we have in-vitro transcribed MVEV and PowV terminal RNA regions and performed their native purification, studied their homogeneity using analytical ultracentrifugation, determined their low-resolution structures, and combined computational modeling to obtain high-resolution structural models. Detailed information on the molecular biology of flavivirus and their life cycle is already available (e.g. [71]). However, there are limited biochemical and biophysical studies of flaviviral nucleic acids, their interactions with host proteins, and the functional role of flaviviral nucleic acids – host protein interactions. Without detailed information on flaviviral nucleic acid tertiary structures, it will be challenging to obtain a complete picture of how flaviviral TRs play a part in viral replication. Recently, it was shown for the West Nile virus that the 3’ stem-loop region undergoes a temperature-dependent genome cyclization, meaning that the higher temperatures in humans facilitate genome cyclization and viral replication, which does not occur in mosquitos due to their decreased body temperature [72]. Potential temperature-based change in viral RNA tertiary structure is an important aspect to investigate further. We demonstrated that to gain high-resolution viral RNA structural details, a combination of SAXS and computational methods is feasible. This approach will help us understand the role of flaviviral tertiary structure in viral replication, through the establishment of a pipeline that allows for 3-dimensional visualization of RNA and RNA-protein complexes. 

## Figures and Tables

**Table 1:** Biophysical parameters of MVEV and PowV non-coding RNAs.

Sample	MVEV 5’TR	MVEV 3’TR	PowV 5’TR	PowV 3’TR
*M_w_* (kDa, sequence)	31.38	31.57	36.92	37.80
Sedimentation coefficient (S)^∇^	4.27 (4.08, 4.46)	4.30 (4.20, 4.41)	4.50 (4.42, 4.55)	4.53 (4.52, 4.53)
*I(0)^#^*	0.0098 ± 4.7 × 10^5^	0.019 ± 1.4 × 10^5^	0.019 ± 6.7 × 10^5^	0.034 ± 8.2 × 10^5^
*q.Rg range*	0.38 – 1.26	0.59 – 1.26	0.34 – 1.26	0.47 – 1.29
*R_g_* (Å)^#^	29.01 ± 0.22	45.72 ± 0.48	34.65 ± 0.19	35.84 ± 0.13
*I(0)^#^* ^∆^	0.098 ± 3.8 × 10^5^	0.018 ± 8.1 × 10^5^	0.019 ± 4.2 × 10^5^	0.034 ± 8.9 × 10^5^
*R_g_* (Å)^∆^	30.16 ± 0.11	46.16 ± 0.18	34.48 ± 0.06	38.39 ± 0.08
*D_max_* (Å) ^∆^	100	150	105	130
**χ**^2^*	~0.78	~0.83	~0.78	~0.80
NSD *	0.71 ± 0.01	0.80 ± 0.02	0.85 ± 0.02	0.72 ± 0.01

The *M_w_* values were calculated using nucleotide sequences. ^∇^- determined using SV-AUC analysis and UltraScan-III package [60]. Sedimentation coefficients obtained following genetic algorithm-Monte Carlo analysis. The measured value represents the mean. Bracketed values represent a 95% confidence interval from Monte Carlo analysis. ^#^ - obtained from Guinier analysis [44]. ^∆^ - determined using *P*(*r*) analysis using the GNOM program [47]. * - values derived from DAMMIN [48] and DAMAVER [50] analysis.

## Supplementary Materials

The following are available online at https://www.mdpi.com/1999-4915/12/2/190/s1. Figure S1: Sequence alignment of 5’ (top panel, first 95 nucleotides) and 3’ (bottom panel, last 101 nucleotides) TRs of Powassan, Murray valley encephalitis, dengue, and West Nile viruses; Figure S2: The lowest energy structures of MVEV and PowV 5’ and 3’ terminal regions are presented in Figure 1.
