# Supplementary material for: Nanoscale Structure Determination of Murray Valley Encephalitis and Powassan Virus Non-Coding RNAs
Source: Viruses. 2020 Feb 8;12(2):190. doi: 10.3390/v12020190 (PMC7077200; doi:10.3390/v12020190)
Supplement: Supplementary file 1 [file viruses-12-00190-s001.pdf]

viruses

MDPI

Article

Nanoscale Structure Determination of Murray Valley Encephalitis and Powassan Virus Non-coding RNAs

Tyler Mrozowich [1], Amy Henrickson [1], Borries Demeler[1,4,5] and Trushar R Patel [1,2,3,5]*

Supplementary Information

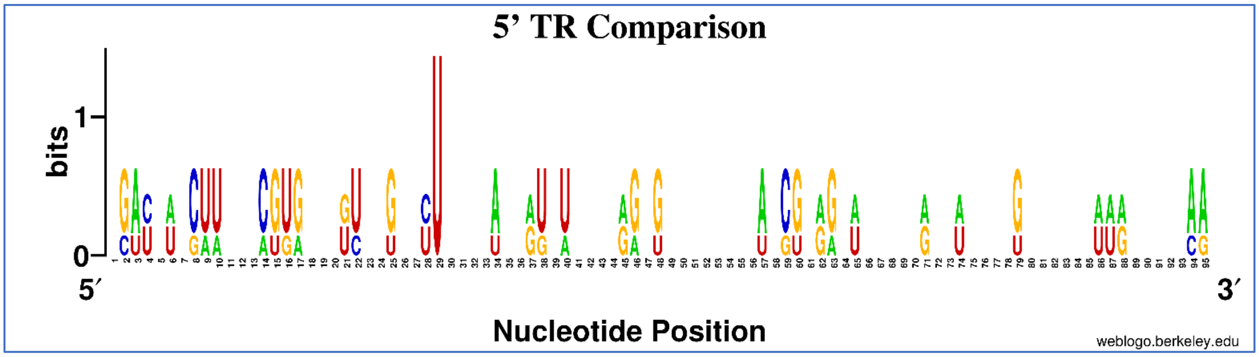

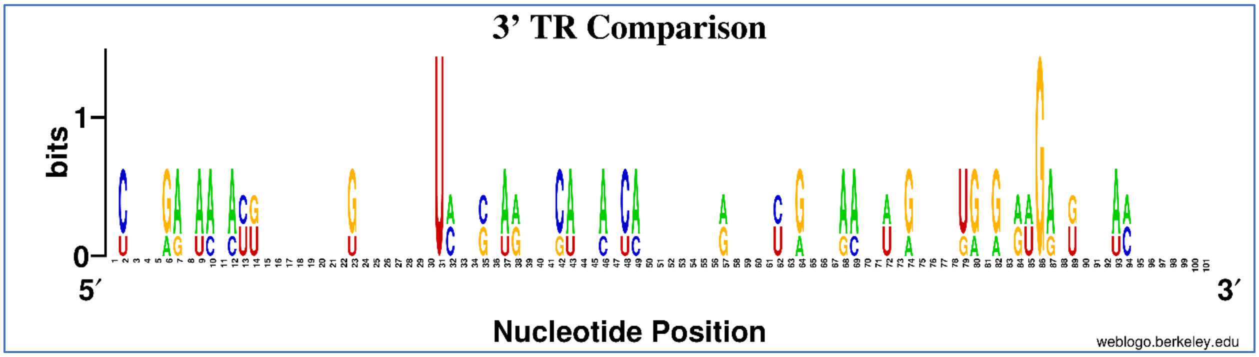

Supplementary Figure 1: Sequence alignment of 5′ (top panel, first 95 nucleotides) and 3′ (bottom panel, last 101 nucleotides) TRs of Powassan, Murray valley encephalitis, dengue, and West Nile viruses. The sequences used for alignment are presented below. The alignment was performed using WEBLOGO (https://weblogo.berkeley.edu/logo.cgi).

>PowV 5'TR
AGAUUUUCUUGCACGUGUGUGCGGGUGCUUUAGUCAGUGUCCGCAGCGUUC
UGUUGAACGUGAGUGUGUUGAGAAAAAGACAGCUUAGGAGAACAAGAGCU

viruses

>MVEV 5'TR
CCUGGGAAAAGACUAGGAGAUCUUCUGCUCUAUUCCAACAUCAGUCACAAG
GCACCGAGCGCCGAACACUGUGACUGAUGGGGGAGAAGACCACAGGAUCU

>DENV 5'TR
GCUUAACGUAGUGCUGACAGUUUUUUAUUAGAGAGCAGAUCUCUGAUGAAC
AACCAACGGAAGAAGACGGGAAAACCGUCUAUCAAUAUGCUGAAACGCGU

>WENV 5'TR
UGACAAACUUAGUAGUGUUUGUGAGGAUUAACAACAAUUAACACAGUGCG
AGCUGUUUCUUAGCACGAAGAUCUCGAUGUCUAAGAAACCAGGAGGGCCCG

3’TR comparison (First 101 nt)

>PowV 3'TR
CCCCCAGGAAACUGGGGGGGCGGUUCUUGUUCUCCCUGAGCCACCACCAUCC
AGGCACAGAUAGCCUGACAAGGAGAUGGUGUGUGACUCGGAAAAACACC

>MVEV 3'TR
CCUGGGAAAAGACUAGGAGAUCUUCUGCUCUAUUCCAACAUCAGUCACAAG
GCACCGAGCGCCGAACACUGUGACUGAUGGGGGAGAAGACCACAGGAUCU

>DENV 3'TR
UCUUGGACUAUAUGCCUUCAAUGAAGAGGUUCAGGAAGGAAGAGGAGUCGG
AGGGGGCCAUUUGGUAAACGUAGGAAGUGAAAAAGAGGCUAACUGUCAGG

>WENV 3'TR
AUGAAGACACAACUUUAGUUGAGGACACAGUACUGUAAAUACUUUAUCAA
UUGUAAAUAGACAAUAUAAGCAUGUAUAUAGGUAUGGGUUUAUAGUGGCA
U

viruses

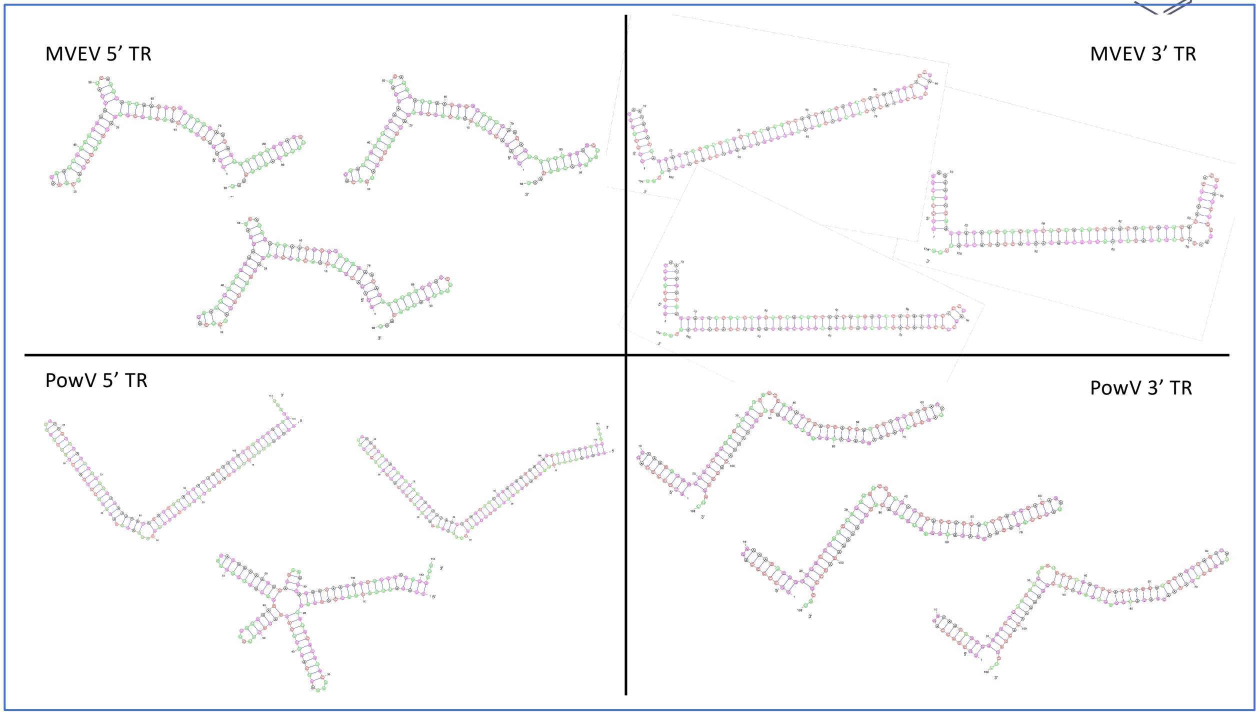

Supplementary Figure 2: The lowest energy structures of MVEV and PowV 5' and 3' terminal regions are presented in Figure 1. This figure illustrates the next three lowest energy structures for each RNA structure.
